# Microvascular Dysfunction in Peripheral Artery Disease: Is Heat Therapy a Viable Treatment?

**DOI:** 10.3390/ijerph18052384

**Published:** 2021-03-01

**Authors:** Cody P. Anderson, Elizabeth J. Pekas, Song-Young Park

**Affiliations:** 1 School of Health and Kinesiology, University of Nebraska at Omaha, Omaha, NE 68182, USA; codypanderson@unomaha.edu (C.P.A.); lizpekas@unomaha.edu (E.J.P.); 2 Department of Biomechanics, University of Nebraska at Omaha, Omaha, NE 68182, USA

**Keywords:** atherosclerosis, endothelial dysfunction, microcirculation, peripheral vascular disease

## Abstract

Peripheral artery disease (PAD) is characterized by the development of atherosclerotic plaques in the lower-body conduit arteries. PAD is commonly accompanied by microvascular disease, which may result in poor wound healing, plantar ulcer development, and subsequent limb amputation. Understanding the mechanisms underlying the development of plantar ulcers is a critical step in the development of adequate treatment options for patients with PAD. Skin is classified into two major components: glabrous and non-glabrous. These skin types have unique microcirculation characteristics, making it important to differentiate between the two when investigating mechanisms for plantar ulcer development in PAD. There is evidence for a microcirculation compensatory mechanism in PAD. This is evident by the maintenance of basal microcirculation perfusion and capillary filling pressure despite a reduced pressure differential beyond an occlusion in non-critical limb ischemia PAD. The major mechanism for this compensatory system seems to be progressive vasodilation of the arterial network below an occlusion. Recently, heat therapies have emerged as novel treatment options for attenuating the progression of PAD. Heat therapies are capable of stimulating the cardiovascular system, which may lead to beneficial adaptations that may ultimately reduce fatigue during walking in PAD. Early work in this area has shown that full-body heating is capable of generating an acute cardiovascular response, similar to exercise, which has been suggested as the most efficient treatment modality and may generate adaptations with chronic exposure. Heat therapies may emerge as a conservative treatment option capable of attenuating the progression of PAD and ultimately impeding the development of plantar ulcers.

## 1. Introduction

Peripheral artery disease (PAD), which affects an estimated 200 million people globally [1], is characterized by the development of atherosclerotic plaques in the peripheral conduit arteries [2,3,4]. The most common manifestation of PAD is claudication, which includes symptoms such as leg pain, cramping, fatigue, pressure, and muscle weakness. These symptoms are often brought on by physical activity and are attenuated by rest [5]. In late-stage PAD, critical limb ischemia may develop, which occurs when tissue perfusion can no longer match the metabolic demand of the tissues below an arterial occlusion at rest. This often results in chronic ischemic pain, ulcers, and ultimately amputation [6]. It has been reported that those with PAD are at an increased risk for developing cerebral artery disease and coronary artery disease [7]. Additionally, patients with PAD are prone to increased rates of depression, which is strongly associated with all-cause mortality [8]. PAD is also independently associated with a 13.9-fold increase in amputation risk, which is further increased if PAD is confounded by microvascular disease [9].

The manifestation of microvascular disease is independent of the development of atherosclerosis in conduit arteries, but microvascular disease has been identified as a common phenomenon in those with PAD [9]. It has been documented that many adaptations occur in cutaneous microcirculation throughout the progression of PAD, including reduced capillary density in late-stage PAD [10]. The presence of microvascular dysfunction/disease in PAD is associated with poor wound healing and ischemic plantar ulcers [9]. The development of ischemic plantar ulcers may be dangerous for patients with PAD because of the potential for subsequent lower limb amputation. Therefore, understanding the mechanisms underlying microvascular dysfunction/disease and resultant ulcers in PAD may be important for the development of better treatments and therapeutics targeted at reducing symptoms and improving quality of life. 

It has been suggested that understanding the different classifications of skin and how they are affected by microvascular dysfunction/disease may be important for the prevention of plantar ulcer development. Two major skin types, glabrous and non-glabrous, have unique structural and microcirculatory characteristics [11], so they may have different responses to ischemic stress and various stimuli, such as heat. Therefore, understanding the characteristics of these skin types and their microcirculatory environments will be crucial for the development of future therapeutics for PAD. 

Although there are no clinically efficient therapies available, heat therapies have recently emerged as a top-priority area of research in the PAD literature. Therapies utilizing heated-water immersion and heated-water exercise have demonstrated promising success [12,13,14,15]. Several benefits for acute and chronic hemodynamics have been reported for heated-water immersion in PAD [12,13]. In addition, we have previously shown that exercise training in heated water promotes hemodynamic benefits, favorable vascular structural changes, and improved quality of life in PAD [14,15]. In fact, exercise in heated water demonstrated relatively higher adherence to the clinically recommended land-based training [15]. The hemodynamic and vascular structural changes associated with heat therapy may lead to adaptations beneficial to cutaneous microcirculation and may serve a role in preventing ischemic ulcers. However, the mechanisms underlying the hemodynamic and structural adaptations due to heat therapy are poorly understood. This review will focus on cutaneous microcirculation in PAD, propose evidence for a microcirculatory mechanism in PAD, explain the effects of heat therapy on hemodynamics in PAD, and discuss how the characteristics of PAD microcirculation may be adapted with heat therapy. 

## 2. Skin Types and Microcirculation in PAD

Cutaneous microcirculation is responsible for perfusing dermal and epidermal cell structures and for full-body thermoregulation [11,16,17]. The majority of cutaneous microcirculation is in the upper horizontal plexus, which resides 1–2 mm deep to the epidermal surface [18]. The upper horizontal plexus is in charge of maintaining a large potential for temperature regulation [18,19]. Importantly, the ultrastructure of cutaneous microcirculation in the upper horizontal plexus is further complicated by whether the region is classified as glabrous or non-glabrous. This skin type classification and its subsequent microcirculatory properties may yield further insight regarding microcirculatory dysfunction and ulcer development in PAD. 

**Non-glabrous microcirculation:** Non-glabrous skin, which contains hair follicles [11], is innervated by both a sympathetic vasoconstrictor and vasodilator system [20]. Several local factors contribute to the control of skin perfusion in non-glabrous skin, such as local temperature changes [21,22]. There is evidence indicating that non-glabrous microcirculation has limited capacity for autoregulation, given that perfusion pressure and skin blood flow changes exhibit a direct relationship [23]. However, non-glabrous skin does contain a unique regulatory feature, which is the venoarteriolar reflex (VAR) [20]. The VAR serves to combat blood pressure fluctuations, which may compensate for the deficiency in autoregulatory capabilities [20]. When orthostatic pressure is altered, specifically when cutaneous venule pressure increases, such as during a change in posture, the VAR, a local sympathetic reflex, is activated and causes vasoconstriction of arterioles, which protects capillary structures against the flux of blood brought about by the changing gravitational potential [24]. 

**Glabrous microcirculation:** Glabrous skin is non-hairy skin found on the plantar aspects of the feet, the palmar sides of the hands, and in various locations on the head [11]. Contrary to non-glabrous skin microcirculation, the glabrous microcirculation is thought to have autoregulatory capabilities but does not contain a VAR [20,25]. A structure entirely unique to glabrous skin is the arteriovenous anastomose (AVA) [26]. AVAs are direct conduits between dermal arterioles and venules, thus AVAs do not contribute to nutritive circulation like that of capillary networks and loops. AVAs contain thick concentric layers of smooth muscle and are densely innervated by sympathetic adrenergic nerves [26]. However, there is no active vasodilator system, so AVAs do not respond to metabolic vasodilators [27]. AVAs are thought to be under central control and are integral components of core temperature regulation [26]. In the thermoneutral temperature zone, the AVAs remain mostly closed, but during periods of extreme heat stress, the AVAs may become completely dilated (via release of sympathetic tone) to allow for increased blood volume closer to the external environment [26]. 

When the microcirculatory network becomes dysfunctional in chronic ischemia (i.e., PAD), the autoregulatory potential and function of VARs and AVAs may be attenuated. Midttun et al. (1997) examined the impacts of orthostatic pressure on glabrous skin microcirculatory characteristics in PAD [25]. Microcirculatory flow was assessed in the great toe at the level of the heart, above the heart, and below the heart. At the level of the heart, it was found that nutritive perfusion in claudicating patients was similar to healthy controls. When the great toe was lowered below the heart, the controls exhibited no change in cutaneous microcirculation perfusion, which was attributed to the absence of a VAR in the glabrous skin of the great toe. The maintenance of perfusion, despite orthostatic pressure changes in the great toe, was primarily attributed to autoregulation. However, claudicating patients with PAD demonstrated an increase in blood flow nearly 1.6x greater than what was measured at the level of the heart [25]. This lack of perfusion control in PAD may indicate attenuated autoregulatory potential in the glabrous microcirculation, considering that flow increased when the orthostatic pressure was increased. 

The hydrostatic hypothesis, which is a proposed mechanism for plantar ulcer development in diabetics, states that ulcer development is caused by edema formation in the skin, due to increased hydrostatic pressures in the capillary beds [28]. While the effects of diabetes on microcirculation are not directly transferable to PAD, the loss of glabrous autoregulation in PAD may contribute to ulcer development in a similar manner as stated by the hydrostatic hypothesis. Without adequate autoregulation, changes in orthostatic pressure would lead to intermittently higher capillary pressures and hydrostatic pressures in the glabrous capillaries of the plantar foot in PAD [24]. It has also been stated that increased capillary hydrostatic pressures lead to an increase in basement membrane thickness [24]. An increase in basement membrane thickness would increase the diffusion distance for oxygen [29] and therefore could contribute to the progressive ischemia, tissue damage, and fibrosis associated with PAD.

Differentiating microcirculation between glabrous and non-glabrous skin will be crucial for understanding cutaneous perfusion and full-body thermoregulation [11,16,17], since glabrous and non-glabrous skin have unique control systems and structures. Studies investigating glabrous skin are useful for elucidating the mechanisms of plantar ulcer formation in PAD because the plantar foot is predominately glabrous [26]. Patients with PAD exhibit reduced capacity to autoregulate perfusion in the glabrous skin during changes in orthostatic pressure, which may be a key mechanism for plantar ulcer development in this population [25].

## 3. Microcirculation in PAD: Endothelial-Dependent and Endothelial-Independent Mechanisms

Patients with PAD exhibit reduced arterial pressures below an occlusion [30,31]. According to Poiseuille’s law, if resistance remains constant, a reduced pressure differential results in reduced flow [32]. However, it has been documented that patients with PAD (non-CLI) preserve both cutaneous microcirculation (flow) [24,25,28,33,34,35,36] and venous filling pressure [28] at rest (Table 1). Thus, to satisfy Poiseuille’s law, there must be a reduction in the resistance of the post-occlusion network to maintain flow [32] when the pressure differential is reduced [30,31]. This may indicate a compensatory mechanism in the vascular network below an occlusion in PAD. A change in arteriole tone, and therefore resistance, could influence microcirculation characteristics and thus it is important to elucidate any compensatory mechanism(s) that may affect microcirculation to better understand plantar ulcer development in PAD.

**Microvascular response to post-occlusive hyperemia (reactive hyperemia):** Post-occlusive hyperemia is a well-accepted assessment to non-invasively evaluate vascular endothelial function, which is one of the major vasodilation mechanisms. Rossi et al. (2005) revealed a potential compensatory mechanism while examining reactive hyperemia in patients with PAD [35]. A pneumatic cuff was applied to the upper thigh to induce acute ischemia of the peripheral tissue. Flow motion waveforms were decomposed into cardiac, respiratory, neurogenic, myogenic, and endothelial contributions [35]. This study reported that neurogenic, myogenic, and endothelial spectral powers were significantly higher in PAD compared to healthy subjects at rest. It was also reported that cardiac spectral power was significantly reduced in PAD compared to healthy subjects. There was no significant difference in respiratory contribution or basal microcirculation blood flux. Following post-occlusion hyperemia, neither group demonstrated a significant increase in endothelial, myogenic, or neurogenic activity, but there was a significant increase in cardiac activity in both PAD and healthy subjects. There was no difference in peak hyperemia between groups, but there was a significant difference in the time to peak hyperemia and the time to recovery after peak hyperemia between PAD and healthy subjects [35]. The previous study [35] suggests a compensatory mechanism in the post-occlusion network employed to maintain perfusion in microcirculation. The decrease in cardiac contribution to flow motion can be explained by the nature of an occlusion. If the pulse wave generated by the heart during systole were to represent a signal generated by the heart, an occlusion would distort the cardiac signal via changes in hemodynamics [35]. Thus, an occlusion would naturally downregulate the cardiac contribution to vasomotion. To compensate for the downregulation of cardiac contribution, the endothelium, smooth muscle, and nervous system activity are upregulated to maintain appropriate flow motion in the post-occlusion network. This compensatory mechanism is also supported by the findings regarding time to peak flux and recovery. Cardiac activity represents the highest-frequency contributor to flow motion [37], thus any reduction in cardiac contribution would decrease the rate at which flow motion could change. Rossi et al. (2005) reported an increased time to peak hyperemia and recovery after hyperemia in subjects with PAD [35], which likely indicates that low-frequency mechanisms of flow motion contribute more than high-frequency mechanisms of flow motion in PAD. Given that patients with PAD maintain normal flow characteristics in cutaneous microcirculation below an occlusion at rest [24,25,28,33,34,35,36], it can be inferred that the upregulation of endothelial, myogenic, and neurogenic activity [35] is adequate enough to maintain flow below an occlusion at rest, despite a decreased pressure differential [30,31].

**Figure 1:**
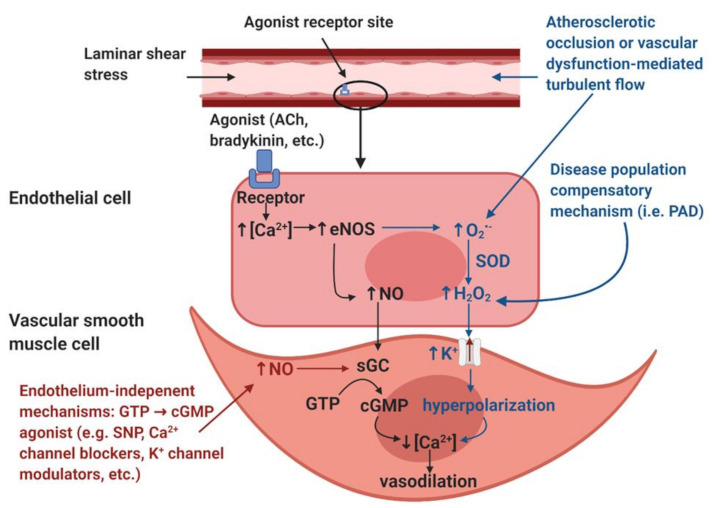
Illustration of the nitric oxide (NO)-mediated endothelial-mediated vasodilation pathway (black), the hypothesized disease population endothelial-dependent compensatory mechanism (blue), and endothelium-independent modulators of vasodilation (red). Image created with BioRender.com.

**Endothelial adaptations and the roles of hydrogen peroxide in vasodilation mechanisms:** Endothelial-dependent vasodilation is positively coupled to blood pressure changes [36] and altered shear stress [38], so the increase in activation of endothelial cells observed by Rossi et al. (2005) [35] appears contradictory, considering that blood pressure and flow is reduced below an occlusion [30,31]. Although it is reported that capillary flow and filling pressure are preserved in PAD [24,25,28,33,34,35,36], flow would be reduced in all downstream blood vessels from the occlusion to the capillary beds. Since shear stress is directly coupled to vessel diameter and blood velocity, any vessels below an occlusion, with reduced flow, would experience a reduction in shear stress, which would result in less nitric oxide (NO), a potent endothelial produced vasodilator (Figure 1). Therefore, anywhere that a reduction in shear stress exists, there should be a decrease in the endothelial mediated NO contribution to vasodilation. 

A possible explanation for the increase in endothelial-dependent vasodilation despite decreased shear stress and blood pressure may be explained by a “vasodilatory agonist switch,” which represents the shift of endothelial mediated vasodilation from nitric oxide to the endothelial derived hyperpolarization factor (EDHF), hydrogen peroxide (H_2_O_2_) [39] (Figure 1). It has been suggested that in states of disease, such as PAD, H_2_O_2_ may become favored over NO to facilitate vasodilation [39]. NO facilitates vasodilation by causing smooth muscle relaxation via calcium regulation, whereas H_2_O_2_ is capable of causing smooth muscle relaxation via hyperpolarization of the smooth muscle membrane [40]. Stress may be able to stimulate the production of H_2_O_2_ in endothelial cells [39]. This would provide an alternative route for the production of vasodilation agonists, independent of shear stress. It is likely that the tissues below an occlusion may be the main producers of inflammatory markers that increase the production of vasodilation agonists [1]. While it is supported that patients with PAD have elevated markers of inflammation [41], elevated H_2_O_2_ production is not directly coupled to PAD [41]. It may be that an agonist switch only occurs in highly progressed stages of PAD. Given that H_2_O_2_ is pro-inflammatory, a positive feedback loop of inflammation would likely result from H_2_O_2_ becoming the main agonist of vasodilation [39]. Whether inflammatory markers are capable of facilitating the production of NO is beyond the scope of this review. 

***NO-mediated vasodilation (black).*** The endothelial layer is stimulated by laminar shear stress and/or signaling molecules such as acetylcholine (ACh) and bradykinin. These stimuli induce an increase in intracellular calcium (Ca^2+^) within the endothelial cells. The increased [Ca^2+^] induces the activation of the calcium-dependent enzyme endothelial nitric oxide synthase (eNOS), resulting in the production of nitric oxide (NO). NO diffuses into the smooth muscle and binds to the receptor soluble guanylyl cyclase (sGC), which induces the accumulation of cyclic guanosine monophosphate (cGMP) from guanosine triphosphate (GTP). The accumulation of cGMP reduces intracellular [Ca^2+^] within the vascular smooth muscle cells and thus induces vasodilation. 

***Disease population compensatory mechanism (blue)*.** In disease populations such as peripheral artery disease (PAD), it is hypothesized that the endothelial-derived hyperpolarization factor (EDHF) hydrogen peroxide (H_2_O_2_) may replace the NO-mediated vasodilatory pathway. Atherosclerotic occlusion or vascular dysfunction-mediated turbulent flow induces the formation of reactive oxygen species, specifically superoxide (O_2_^•−^). In addition, increased O_2_^•−^ production is also thought to be partially mediated by eNOS uncoupling, a condition in which eNOS produces O_2_^•−^ as opposed to NO. Dismutation by superoxide dismutase (SOD) or spontaneous dismutation occurs within the endothelial cell, in which O_2_^•−^ and hydrogen ions produce H_2_O_2_. H_2_O_2_ diffuses into the vascular smooth muscle cell, which stimulates potassium (K^+^) channels, resulting in an efflux of K^+^ ions and resultant hyperpolarization of the vascular smooth muscle cells. This hyperpolarization results in a reduction in intracellular [Ca^2+^], which induces vasodilation.

***Endothelium-independent modulators of vasodilation (red).*** Endothelium-independent mechanisms may also induce vascular smooth muscle relaxation. These pathways bypass the endothelium and include GTP → cGMP agonists such as sodium nitroprusside (SNP), Ca^2+^ channel blockers, K^+^ channel modulators, etc. The accumulation of cGMP from GTP will reduce intracellular [Ca^2+^] in the vascular smooth muscle cell, thus inducing vascular smooth muscle relaxation.

**Vasodilation responses to heating:** Local heating applications are another way to examine the reactivity of cutaneous microcirculation. Parshakov et al. (2016) examined the reactivity of PAD cutaneous microcirculation to local heating [3]. Temperature flux waveforms on the plantar surface of the great toe, isolating endothelial, myogenic, and neurogenic contributions to the local heating response were assessed [3]. Previous works have demonstrated a positive correlation between temperature waveforms and blood flow waveforms in the frequency domains related to endothelial, myogenic, and neurogenic activity [42]. Parshakov et al. reported that during heating, the relative contributions of the endothelium, smooth muscle, and nervous system were downregulated in PAD compared to healthy controls [3].

These findings initially seem contradictory to the work of Rossi et al. (2005), which demonstrated an upregulation of endothelial, myogenic, and neurogenic activity in PAD [35], but on further analysis, these findings are complementary. These findings can be explained by the operation limits of living organisms, which suggest that the variability of vasomotion has an upper and lower bound, between which it operates to maintain vascular homeostasis. If the mechanisms responsible for vasodilation reside at an elevated steady state [35], the maximum potential for vasodilation would be reduced because the system would continue to operate under the original physiological limits. If the potential to respond to a stimulus is attenuated, this would present as a downregulated response when a stimulus is imposed on the system. Therefore, the attenuated vascular reactivity to a heating stimulus observed by Parshakov et al. [3] seems to be explained by the elevated vasodilator steady state documented by Rossi et al. [35]. 

**Receptor mediated vasodilation responses:** Acetylcholine (Ach), a muscarinic receptor agonist, is considered to be an endothelial-dependent vasodilator, and sodium nitroprusside (SNP), an NO donor, is considered to be an endothelial-independent vasodilator (smooth muscle function) [40]. Thus, these two receptor agonists for endothelial-dependent and -independent vasodilation may elucidate the health of both the endothelium and smooth muscle. In two separate experiments, Rossi et al. (2002, 2005) examined maximal vasodilation in cutaneous microcirculation below an occlusion in patients with PAD via pharmacological-mediated vasodilation [34,35]. Through multistage vasodilation, it was found in both experiments that at mid to high stages, the vasodilatory response was significantly attenuated in PAD compared to healthy controls with both Ach and SNP administration [34,35]. The function representing the vasodilatory response to Ach administration resembled the upper end of a sigmoidal curve in the PAD group, indicating a limit, but increased without bound in the healthy controls. Limits were not clearly visible in the vasodilatory response to progressive SNP administration [34,35]. 

These results [34,35] further complement the notion of compensatory mechanisms existing below an occlusion in PAD. It was previously mentioned that patients with PAD exhibit a blunted response to local heating [3] and this was interpreted as a relationship between physiological limits and an elevated steady state [35], rather than damage to the vasodilatory mechanisms themselves. This reasoning can also be applied to the PAD vasodilatory response to the administration of Ach and SNP. In both experiments by Rossi et al. (2002, 2005), the PAD group achieved clear vasodilation limits, while control subjects did not demonstrate a vasodilatory limit [34,35]. Rather than attribute the observed limits [34,35] to endothelial dysfunction, this review suggests that these limits may indicate the interaction between an elevated resting vasodilation steady state and a constant physiological limit.

**Figure 2:**
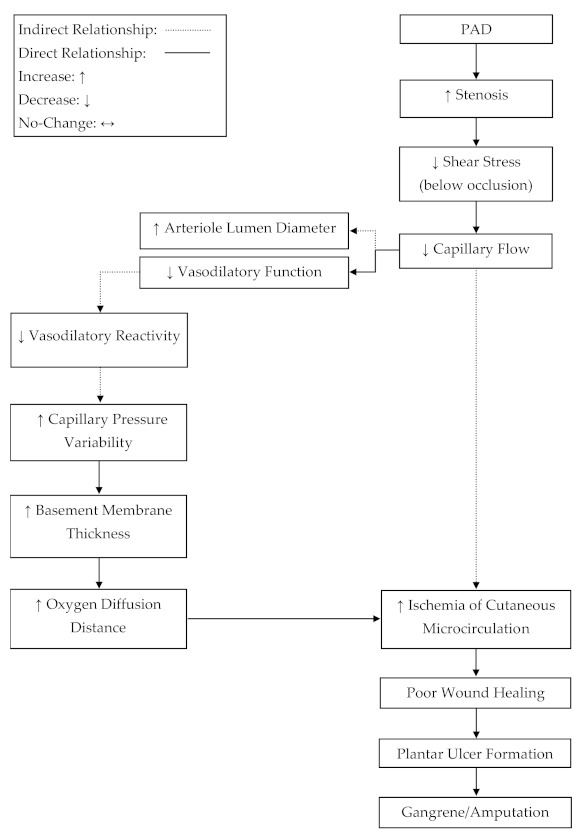
A proposed mechanism for progressive tissue ischemia below an occlusion in peripheral artery disease (PAD). The progressive narrowing of the conduit arteries and the reduced reactivity of arterioles, due to plaque development, may lead to ischemia-related, plantar ulcers and subsequent amputation.

Blood flow and venous filling pressure are preserved in cutaneous microcirculation in patients with PAD [24,25,28,33,34,35,36]. In order to satisfy Poiseuille’s law, resistance below an occlusion must decrease to maintain flow in the wake of a decreased pressure differential [32]. A potential mechanism, which may lead to reduced resistance in the circulatory network below an occlusion, may be increased arteriole vasodilation. This review suggests that the increase in vasodilation steady state [35], limits to relative vasodilatory responses [34,35], and the preservation of cutaneous microcirculation blood flow [24,25,28,33,34,35,36] and venous filling pressure [28] are indicative of a compensation mechanism for which vasodilation is increased below an occlusion to decrease resistance and satisfy Poiseuille’s law [32]. In addition to this evidence, it has been observed that arterioles in patients with PAD, suffering from critical limb ischemia (CLI), are near maximally dilated at rest [43]. This may suggest a progressive compensation mechanism for which the vasodilation steady state below an occlusion is gradually upregulated in parallel to the progressive narrowing of the upstream occlusion, resulting in elevated dilation at rest. Developing an understanding of a compensatory mechanism, where basal arteriole tone in microcirculation is progressively shifted towards dilation, could be important for elucidating the progression of plantar foot ulcers in PAD (Figure 2).

## 4. Clinical Applications of Heat Therapy

Heat therapy, or the use of an external heating source to increase local or systemic body temperatures, has been emerging as an efficient intervention for patients with PAD. Heat therapies include a broad spectrum of methods, such as heated-water submersion or external heating via water circulating trousers. In general, during exercise, cardiac output increases and peripheral vascular resistance decreases due to local vasculature vasodilation and autoregulation [29]. Similarly, when core temperature increases, cardiac output increases and peripheral resistance decreases [17], due to changes in arteriole tone and the tone of AVAs in the cutaneous circulation. Independently, both stimuli elicit similar outcomes on hemodynamics [12,13], but unlike exercise alone, the response to heat does not rapidly induce fatigue in PAD [44]. This may make it possible to maintain the beneficial hemodynamics, typically achieved through exercise, for longer, with increases in core temperature. Intermittent increases in core temperature have been shown to induce arterial adaptations in healthy populations [45], but the benefits of intermittent full-body heating on PAD progression and plantar ulcer development have yet to be elucidated. However, several groups have recently demonstrated beneficial hemodynamic and vascular changes in response to both acute and chronic heat therapies. 

Acute heat therapy has demonstrated benefits regarding circulating endothelial cell health biomarkers and hemodynamics in patients with PAD. Neff et al. (2016) utilized leg thermotherapy to examine the impacts of acute heating on circulating endothelial cell health biomarkers and hemodynamics in PAD [46]. They found that endothelin-1 (−60%), a potent vasoconstrictor, as well as systolic (SBP) and diastolic (DBP) blood pressure (SBP: −11 mmHg, DBP: −6 mmHg) were significantly reduced after acute heating. The authors also noted a significant increase in blood velocity (+70%) and blood flow (+100%) in the popliteal artery, which may be an important stimulus for inducing improvements in endothelial function and long-term vascular adaptations. In addition, Thomas and colleagues (2017) showed that following an acute heating bout, shear rate (+260%) and blood flow (+226%) in the popliteal artery were significantly increased, while mean arterial pressure (−20%) and pulse wave velocity (−0.3 m/s) were significantly reduced in patients with PAD [12].

Chronic heat therapies have been used in both animal models of PAD and patients with PAD. Roseguini’s group has explored the impacts of chronic heat therapy in murine models of chronic hindlimb ischemia [47,48]. Kim et al. (2019) found that muscular mass (+7%) in the ischemic limb was greater following heat therapy compared to control [47]. In a similar investigation, Kim et al. (2020) found that in addition to preserving muscle mass, heat therapy treatment may also be beneficial for improving body composition in an obese murine model of hindlimb ischemia. However, the authors noted that there were no improvements in capillary or collateral growth in response to the chronic heat therapy [48]. Overall, Kim et al. (2019 and 2020) revealed that chronic heat therapy may elicit benefits at the muscular level, which may be partially attributed to the increase in core temperature. It is important to note that these findings may not directly translate to human research and warrant further investigation.

Human studies have also revealed that chronic heat therapy may be capable of eliciting benefits for patients with PAD. Akerman and colleagues (2019) investigated the differences in basal hemodynamics in response to either a 12-week heat therapy program (HT) or a 12-week supervised exercise program (EP) [13]. Despite significant improvements in maximal walking distance (HT: +47 m, EP: +35 m) and pain-free walking distance for both groups (HT: +40 m, EP: +46 m), there were no significant differences between conditions. A similar trend occurred for vascular endothelial growth factor (VEGF): concentrations significantly increased ~60% from baseline for both groups, yet no significant differences between the two groups were noted. Both groups demonstrated significantly reduced basal systolic blood pressure, but remarkably, the heat therapy group demonstrated an even greater reduction [13]. These findings indicate that heat therapy may be capable of eliciting adaptations, similar to exercise training, in patients with PAD. Additionally, Monroe et al. (2020) investigated the impacts of chronic lower-limb heating on walking ability, vascular reactivity, and blood endothelial cell health biomarkers in PAD compared to healthy control [49]. There were no changes in 6 min walking distance in either group after the intervention. Neither group experienced a significant change in peak blood flow through the popliteal artery during post-occlusive hyperemia. In addition, cutaneous blood conductance was not significantly different after the intervention. Systolic and diastolic blood pressure remained unchanged. However, the heat therapy group exhibited significantly lower levels of endothelin-1 (−0.4 pg/mL) compared to the sham group [49]. In addition to these objective measurements, Monroe et al. reported a subjective metric, which measured perceived thermal comfort on a 9-point scale (−4 being very cold and 4 being very hot). The investigators found that with circulating water at 48 degrees Celsius, the patients with PAD reported their perceived thermal comfort to be between 2 (warm) and 3 (hot), which demonstrates that the heat therapy was tolerable for the investigated patients with PAD. These acute and chronic heat therapy studies both show that lower-limb heating manipulates circulating endothelial cell health biomarkers [46]. The reduction in endothelin-1 concentration may be beneficial, considering that Parshakov et al. (2016) demonstrated that endothelin-1 concentrations are negatively associated with endothelial mediated vasodilation [3].

The combined effects of exercise and full-body heating have also been recently investigated. Park et al. (2020) examined the effects of a heated-water exercise (HW) protocol and a dry-land exercise (LB) protocol (treadmill training) in patients with PAD [15]. Subjects allocated to both groups participated in their mode of exercise for 12 weeks [15]. Both the heated-water and dry-land exercise groups experienced significant reductions in blood pressure (HW: −8 mmHg, LB: −6 mmHg), resting heart rate (HW: −4 bpm, LB: −2 bpm), and pulse wave velocity (HW: −1.2 m/s, LB: −1 m/s) (a marker of arterial stiffness) compared to baseline. However, the heated-water exercise group showed an even greater reduction in these metrics compared to the land-based group [15]. These results indicate that while both heated-water and dry-land exercise elicit hemodynamic changes and structural changes to the vasculature in PAD, heat and exercise together may be a more potent stimulus for adaptation. Since exercise intensities were matched between groups, this study also indicates that heat may be independently capable of eliciting positive adaptations in PAD. 

**Figure 3:**
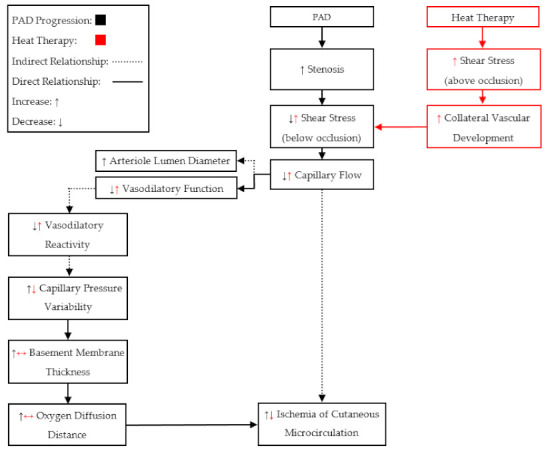
A proposed mechanism for how heat therapy may attenuate the progressive ischemia that is characteristic of peripheral artery disease (PAD). Heat therapy may generate beneficial arterial adaptations, leading to improved arterial function and reduced tissue ischemia.

In summary, the findings from these groups show that both acute and chronic heat therapy may induce beneficial adaptations for patients with PAD (Figure 3). Acute therapy demonstrated several benefits for circulating endothelial cell health biomarkers as well as hemodynamics in patients with PAD. Chronic therapies in both animal models and patients with PAD demonstrated improvements in markers of endothelial health, maximal walking distance, time to claudication, muscular force and mass preservation, and some benefits for hemodynamics. Importantly, the combination of heat therapy with exercise training demonstrated improvements in hemodynamics and arterial health, which suggests that exercise training in heated water may be a more potent stimulus for adaptation compared to land-based exercise training. Additionally, these findings indicate that heat therapy may be capable of attenuating endothelial dysfunction by reducing the concentration of the nitric oxide antagonist, endothelin-1. However, the capability of heat therapies to improve nitric oxide bioavailability, by reducing endothelin-1 concentrations, in patients with PAD, requires further investigation. Additionally, as we discussed above, most of the studies utilizing heat therapies increase core temperature in PAD, yet there are few studies that examine the effects of local heating without increases in core temperature on vascular function, endothelial cell health biomarkers, and walking capacity. Therefore, studies that compare the effects of local heat therapy (no increase in core temperature) vs. full-body heat therapy (increase in core temperature) on vascular response/adaptation and walking performance in PAD are warranted.

We should also notice that the previous studies (Table 2) utilized heat therapies in early (Fontaine I–II) and middle stages (Fontaine III) of PAD. Therefore, the vascular and skeletal muscular responses to heat therapies in late stages (Fontaine > III) of PAD may be different, due to the more severe chronic tissue damage. Patients in late stages of PAD (Fontaine > III) commonly present with ischemic pain at rest, which may attenuate the microcirculation benefits of heat therapy. In late-stage PAD, the vascular structure may be maximally dilated due to the proposed compensatory mechanisms [43]: meaning that local autoregulation would no longer be capable of manipulating perfusion. Therefore, only an increase in cardiac output would be able to perpetuate blood flow to the tissue below an occlusion. It has been shown that an increase in core temperature increases cardiac output in PAD, similar to exercise [12,13], but an increase in body temperature also induces the release of centrally controlled AVAs in the glabrous skin [26]. Therefore, the redistribution of blood flow through non-nutritive AVAs, during full-body heating, may exacerbate the inadequate perfusion to tissue below an occlusion. Additionally, it has been suggested that peripheral neural function and conduction velocity are reduced in late stages of PAD [1,50,51], which potentially attenuates AVA responsiveness to central control and may cause a further complication in the glabrous microcirculation. However, the capability of peripheral nerves to adapt as a result of heat application in late stages of PAD warrants further investigations and is beyond the scope of this review.

## 5. Clinical Implications

PAD is prevalent worldwide [1] and is associated with deleterious health outcomes, including limb amputation [9]. Tissue below an occlusion becomes progressively ischemic, which leads to an inability to heal cutaneous wounds [9]. The development of ischemic ulcers on the plantar aspects of the feet is therefore a significant health risk for those suffering from symptomatic PAD. Understanding the different types of cutaneous microcirculation, and how they are affected by PAD, is an essential step towards the development of treatment paradigms to attenuate and prevent plantar ulcers. 

Based on our work and previous literature from others, a microcirculation compensatory mechanism likely exists in the networks below an occlusion in PAD [35]. This is evident by the preservation of cutaneous microcirculation flow at rest [24,25,28,33,34,35,36], wavelet analysis of flow motion waveforms [35], reactivity tests [3,34,35], and qualitative assessments of arteriole tone [43]. Due to the reduced pressure differential below an occlusion [30,31], and in order to satisfy Poiseuille’s law [32], vasodilatory tone is likely increased in arterioles below an occlusion to maintain adequate flow to the peripheral tissues [43]. This compensatory mechanism may be a factor contributing to the reduced autoregulatory capabilities observed in PAD microcirculation [25]. Thus, it is important to observe this compensatory mechanism as a potential contributor to ischemic plantar ulcer development.

Heat therapy has been shown to safely and effectively generate cardiovascular adaptations in PAD as well as reduce pain and improve overall physical function [13,14,15]. It is likely that in early-stage PAD, heat treatments may delay ulcer development via the production of collaterals to bypass arterial occlusions [38]. In late-stage PAD, the release of AVA tone, due to increased core temperature, may be acutely negative for cutaneous microcirculation flow, but may induce collateral growth [38]. The ability for patients in late-stage PAD to develop collaterals and the responsiveness of AVAs to central governance during late-stage PAD have yet to be elucidated. Heat therapy may therefore be an adequate preventative treatment option for delaying the onset of plantar ulcers, due to adaptations that heat therapy may generate in the cardiovascular system and glabrous microcirculation. 

## 6. Conclusions

Future research should focus on elucidating how full-body heating may yield adaptations to PAD macro- and microcirculation. In order to develop appropriate heat treatment paradigms, it is critical to understand the responsiveness of PAD blood vessels to thermal stimuli. The thermal stimuli should be fully characterized in terms of mode, duration, intensity, and frequency. As well, it may be important to standardize methods within the field, so that results are more comparable. In addition, it may also be important to consider utilizing protocols that include greater mechanistic investigation in response to heat therapies, such as the investigation of blood biomarkers, angiogenesis, and changes within the mitochondrial environment for patients with PAD. Heat therapies should also be vetted to ensure that chronic exposure is safe. Since high core temperatures may influence immune function, it is important to elucidate the consequences of heat therapy on metrics of inflammation. Hoekstra and colleagues (2021) demonstrated that lower-body heating and full-body heating were both capable of increasing IL-6 levels acutely [52]. Importantly, Gardner et al. (2014) showed that patients with PAD, when compared to age- and-comorbidity matched controls, exhibit similar levels of basal inflammation as controls [41]. This may suggest that PAD alone is not independently associated with increased inflammation. Despite these findings, the effect of local and systemic heating on the inflammatory response in patients with PAD is not well understood and requires further investigation. A longitudinal approach should be undertaken to understand how the responsiveness of PAD vasculature to thermal stimuli changes as a function of disease progression. Additionally, to reduce amputation risk and improve the functional quality of life in patients with PAD, microcirculation studies should be focused on the adaptability of the glabrous microcirculation located on the plantar aspects of the feet. Understanding the adaptability of the glabrous skin in PAD is a critical step in attenuating and preventing plantar ulcer development and subsequent amputation. 

## Figures and Tables

**Table 1:** Basal cutaneous capillary perfusion and pressure are preserved in non-critical limb ischemia (CLI) peripheral artery disease (PAD) patients compared to controls. *Level of Disease* is based on the Rutherford and Fontaine classification systems, which classify the progression of PAD into four major categories: Stage I is asymptomatic, Stage II is characterized by intermittent claudication, Stage III is characterized by rest pain, and Stage IV is characterized by tissue loss and ischemic ulcer development.

Study	Measurement Tool	Measurement Location	Level of Disease	Outcomes
Cisek et al. [24]	Laser Doppler Flowmetry	Plantar aspect of great toe	Stage II	↔ Basal perfusion
Rossi et al. [34]	Laser Doppler Flowmetry	Forearm and leg	Stage II	↔ Basal perfusion
Husmann et al. [33]	Laser Doppler Flowmetry	Plantar aspect of great toe	Stage I–III	↔ Basal perfusion
Rossi et al. [35]	Laser Doppler Flowmetry	Dorsum of the foot and forearm	Stage II	↔ Basal perfusion
Midttun et al. [25]	Heat Washout and 133-Xenon Washout	Plantar aspect of great toe	Stage II	↔ Basal perfusion
Graaff et al. [30]	Laser Doppler Flowmetry	Nail fold of hallux, ankle, toe	Stage II–IV	↔ Capillary pressure
Urbanicic et al. [36]	Laser Doppler Flowmetry	Medial malleolus	N/A	↔ Basal perfusion

**Table 2:** Characteristics of the heat therapy studies in patients with peripheral artery disease (PAD) and chronic hindlimb ischemia murine models. The asterisk (*) indicates animal studies. (VEGF—vascular endothelial growth factor; SBP— systolic blood pressure; DBP—diastolic blood pressure; RHR—resting heart rate; PWV—pulse wave velocity; EDT-1—endothelin-1; PBV—popliteal blood velocity; 6MWD—6 minute walk distance; maxPBF—maximum popliteal artery blood flow; maxBC—maximum blood conductance; antPF—antegrade popliteal flow; HR—heart rate).

Study	Modality	Temp. (°C)	Core Temp. Δ (°C)	Exposure Time	Exercise	Outcomes
Akerman et al. [13]	Water	39	1.0	20–30 min; 3–5×/week; 12 weeks	Yes	↑ VEGF, ↓ SBP
Park et al. [15]	Water	30	N/A	60 min; 4×/week; 12 weeks	Yes	↓ SBP, ↓ DBP, ↓ RHR, ↓ PWV
Neff et al. [46]	Heated Trousers	48	0.9	90 min	No	↓ EDT-1, ↓ SBP, ↓ DBP, ↑ PBV
Monroe et al. [49]	Heated Trousers	48	~0.3	90 min; 6 weeks	No	↔ 6MWD, ↔ maxPBF, ↔ maxBC, ↔ SBP, ↔ DBP, ↓ EDT-1
Thomas et al. [12]	Water	42	1.8	30 min	No	↑ antPF, ↓ HR, ↓ SBP
Kim et al. [47] *	Water	37, 39, 41	1.6–4.7	30 min; 6×/week; 3 weeks	No	↑ Muscle force, ↓ Muscle mass
Kim et al. [48] *	Water	39	~3.0	30 min; >3 weeks	No	↓ Fat mass, ↔ Capillary growth

## Data Availability

Not applicable.

## Abbreviations

Ach, acetylcholine; AVA, arteriovenous anastomoses; CLI, critical limb ischemia; DBP, diastolic blood pressure; EDHF, endothelial derived hyperpolarization factor; EP, exercise protocol; H_2_O_2_, hydrogen peroxide; HT, heat therapy; HW, heated-water exercise; LB, dry-land based exercise; NO, nitric oxide; PAD, peripheral artery disease; SBP, systolic blood pressure; SNP, sodium nitroprusside; VAR, venoarteriolar reflex; VEGF, vascular endothelial growth factor.
